# Portal vein resection and reconstruction with artificial blood vessels is safe and feasible for pancreatic ductal adenocarcinoma patients with portal vein involvement: Chinese center experience

**DOI:** 10.18632/oncotarget.20847

**Published:** 2017-09-12

**Authors:** Zhi-Bo Xie, Ji-Chun Gu, Yi-Fan Zhang, Lie Yao, Chen Jin, Yong-Jian Jiang, Ji Li, Feng Yang, Cai-Feng Zou, De-Liang Fu

**Affiliations:** ^1^ Department of Pancreatic Surgery, Pancreatic Disease Institute, Huashan Hospital, Shanghai Medical College, Fudan University, Shanghai 200040, China; ^2^ Department of Plastic & Reconstructive Surgery, Shanghai Ninth People's Hospital, School of Medicine, Shanghai Jiao Tong University, Shanghai 200011, China

**Keywords:** pancreatic ductal adenocarcinoma, portal vein involvement, pancreaticoduodenectomy, portal vein resection, artificial blood vessels

## Abstract

Evidence shows that portal vein resection (PVR) increase the resectability but does little benefit to overall survival in all pancreatic ductal adenocarcinoma (PDAC) patients. But for patients with portal vein involvement, PVR is the only radical choice. But whether the PDAC patients with portal vein involvement would benefit from radical pancreaticoduodenectomy with PVR or not is controversial. All 204 PDAC patients with portal vein involvement were enrolled in this study [PVR group, n=106; surgical bypass (SB) group, n=52; chemotherapy group, n=46]. Overall survival and prognostic factors were analyzed among three groups. Moreover, a literature review of 13 studies were also conducted. Among 3 groups, patients in PVR group achieved a significant longer survival (median survival: PVR group, 22.83 months; SB group, 7.26 months; chemotherapy group, 10.64 months). Therapy choice [hazard ratio (HR) =1.593, 95% confidence interval (CI) 1.323 to 1.918, *P*<0.001], body mass index (HR=0.772, 95% CI 0.559 to 0.994, *P*=0.044) and carbohydrateantigen 19-9 (HR=1.325, 95% CI 1.064 to 1.651, *P*=0.012) were independent prognostic factors which significantly affected overall survival. Pancreaticoduodenectomy combined with PVR and reconstruct with artificial blood vessels is a safe and an appropriate therapy choice for resectable PDAC patients with portal vein involvement.

## INTRODUCTION

Pancreatic ductal adenocarcinoma (PDAC) is one of the most lethal malignant tumors worldwide [1]. The incidence rate of PDAC is increasing in China nowadays [2]. PDAC is a devastating malignant disease and associated with a rather poor prognosis. Median survival of PDAC patients is 3 to 6 months and 5-year survival rate is less than 6% [3–6].

For PDAC patients, pancreatic surgery currently provides the only chance of cure or long-term survival [7]. However, most patients are not candidates for surgical resection due to distant metastasis or vascular involvement at the time of diagnosis [8]. Vascular involvement used to be a contradiction for PDAC surgery because patients with vascular involvement have a high risk of systemic metastasis possibility [9]. With improvements in surgical technique, anesthesia, and critical care support, there has been renewed interest in vascular resection for isolated involvement of the portal vein (PV) in resectable PDAC [10]. PV resection (PVR) has been proved to be safe, however, the use of PVR is still debatable. Many studies claim that PVR increase the resectability of PDAC, but do little benefit to survival [11–13]. Nevertheless, a newly published meta-analysis find out that pancreatic resection with PVR are associated with increased postoperative mortality, higher rates of non-radical surgery and worse survival [14].

Interestingly, we find there is no exact guideline therapy for PDAC patients with PV involvement. According to TNM classification, PV involvement was classified as T_3_ (tumor stage II). Almost all studies enrolled T_3_ adenocarcinoma patients for PVR, while there were still some studies enrolled T_1-2_ and T_4_ [15, 16], even with M_1_ adenocarcinoma patients as the candidates for PVR [11, 17]. As for the patients without PVR, tumor stage vary from I to IV [11, 18–22]. Based on such incomparable baseline characteristics, we could not find out the appropriate therapy for patients with PV involvement.

Patients with PV involvement are associated with a late tumor stage and a worse prognosis compared with patients without PV involvement. The difference among the chosen study population may conclude an improper result. For PDAC patients with PV involvement, the only choice for cure is pancreatic surgery with PVR when the tumor is resectable. With regard to PDAC patients with unresectable tumors, chemotherapy may be a good choice, and surgical bypass (SB) may be another choice to improve life quality when obstructive symptoms occurs. Thus which therapy will get the most benefit for PDAC patients with PV involvement still needs further consideration.

Moreover, venous end to end anastomosis is the most usual reconstruction method during PVR procedures [7, 11, 20, 21]. Remaining tumor cells on the venous stump may help to produce distal metastasis. Artificial blood vessels, are composed of viable tissue represent the ideal vascular graft. Compared with self-venous, compliance, lack of thrombogenicity, and resistance to infections as well as the ability to heal, remodel, contract, and secrete normal blood vessel products are theoretical advantages of artificial blood vessels [23, 24]. With the artificial blood vessels, tumor cell would not be easy to adhere, penetrate and develop distal metastasis.

Based on above concerns, we enrolled all PDAC patients with PV involvement. Performing PVR and using artificial blood vessels for venous reconstruct. We aim to find out whether PVR and reconstruct with artificial blood vessels would benefit for these PDAC patients and to search and provide a proper therapy for PDAC patients with PV involvement.

## RESULTS

### Characteristics of the study population

From 2010 to 2015, 1382 potential eligible PDAC patients were enrolled in this study. Altogether 209 PDAC patients were found to satisfy the inclusion and exclusion criteria (PVR group, n=111; SB group, n=50; chemo group, n=46). However, three PDAC patients in PVR group were found liver metastasis during intraoperative exploration. They underwent SB procedure and were excluded from our study. Another two PDAC patients in PVR group were found that primary tumors were without the ability of reconstruction, and underwent SB procedure thus were divided into SB group. Finally 204 eligible PDCA patients were divided into PVR group (n=106), SB group (n=52) and chemo group (n=46). At baseline, compared with the patients in PVR and SB group, patients in chemo group are more likely to have lower serum ALB levels and a significantly higher serum level of carbohydrateantigen (CA) 19-9 and CA 50 (Table 1).

**Table 1:** Baseline characteristics of the enrolled patients

Index	PVR Group (n=106)	SB Group (n=52)	Chemo Group (n=46)	P value
Male, n (%)	65, 61.3%	35, 67.3%	28, 60.9%	0.732
Age, years	61.45 ± 9.19	61.87 ± 10.62	60.30 ±18.31	0.804
Body mass index, kg/m^2^	22.26 ± 2.79	21.68 ± 2.81	21.92 ± 3.65	0.511
Diabetes, n (%)	19, 17.9%	6, 11.5%	5, 10.9%	0.400
Leukocyte, 10^9^/L	5.65 ± 1.71	5.71 ± 1.83	6.15 ± 2.52	0.334
Albumin, g/L	40.10 ± 3.71	38.81 ± 4.79	36.93 ± 5.14	<0.001
Total bilirubin, μmol/L	12.21 (11.00 – 32.73)	12.00 (9.93 – 23.63)	6.60 (5.28 – 12.03)	0.218
Alanine Transaminase, U/L	37.50 (18.00 – 101.00)	33.50 (14.25 – 67.75)	29.00 (15.00 – 49.25)	0.054
CEA, μg/L	3.34 (1.97 – 6.95)	3.29 (2.10 – 8.75)	3.00 (2.08 – 4.66)	0.383
CA 125, U/ml	42.27 (25.51 – 78.92)	40.04 (26.06 – 52.00)	39.00 (21.63 – 52.00)	0.127
CA 19-9, U/ml	116.25 (36.00 – 375.480)	380.55 (49.88 – 816.30)	441.50 (96.25 – 683.50)	<0.001
CA 50, U/ml	34.50 (10.63 – 73.20)	34.50 (23.00 – 71.25)	62.25 (25.00 – 170.98)	<0.001
Tumor diameters, cm	4.00 ± 1.31	5.56 ± 2.10	/	0.304
Tumor stage				
Stage IIA, n (%)	51, 48.0%	/	/	/
Stage IIB, n (%)	55, 52.0%	/	/	/
Time, minutes	478.14 ± 93.78	213.50 ± 89.73	/	<0.001
Blood loss, ml	600.00 (487.50 – 544.25)	120.00 (75.00 – 240.00)	/	<0.001
R1 resection, %	5, 4.7%	/	/	/
Hospital stay, days	21.02 ± 8.78	12.37 ± 2.62	9.78 ± 3.73	<0.001

Abbreviation: CA= Carbohydrateantigen, CEA= Carcinoembryonic antigen, PVR=Portal vein resection, SB=surgical bypass.

### Overall survival and related risk factors

**Figure 1:**
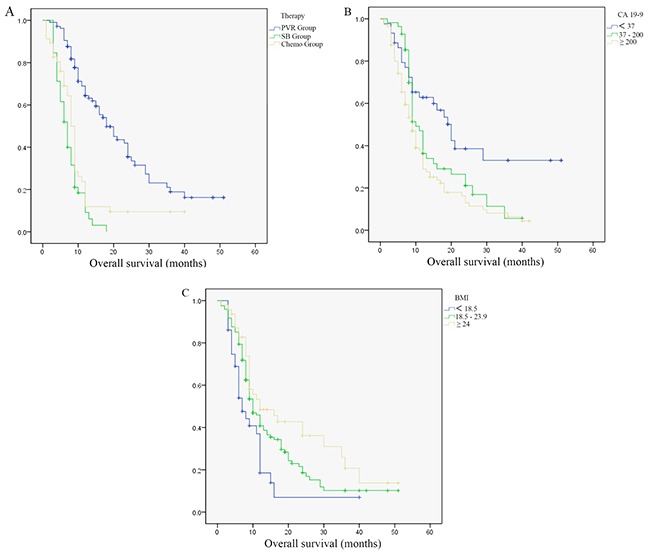
Survivals difference in all pancreatic ductal adenocarcinoma patients **(A)** Survivals difference in patients with different therapy. **(B)** Survivals difference in patients with different carbohydrateantigen 19-9 levels. **(C)** Survivals difference in patients with different body mass index.

In our study, median survivals for patients with PVR, SB and chemotherapy were 22.83 months, 7.26 months and 10.64 months respectively. Among which, patients with PVR had a significantly longer survival (PVR *vs.* SB, *P*<0.001; PVR *vs.* Chemo, *P*<0.001). Patients in chemotherapy group seemed to have better survivals than patients in SB group, but the difference was not significant (*P*=0.064). Univariate and multivariate regression analysis found therapy choice [hazard ratio (HR) =1.593, 95% confidence interval (CI) 1.323 to 1.918, *P*<0.001], body mass index (BMI) (HR=0.772, 95% CI 0.559 to 0.994, *P*=0.044) and CA 19-9 (HR=1.325, 95% CI 1.064 to 1.651, *P*=0.012) could significantly affect overall survival (OS) (Figure 1, Table 2).

**Table 2:** Multivariate analysis of risk factors related with overall survivals in all patients

Risk factors	Univariate analysis			Multivariate analysis			Overall survival	
	HR	95% CI	P value	HR	95% CI	P value	Mean ± SD, months	P value
Therapy								
Group 1: PVR	1.711	1.427–2.050	<0.001	1.593	1.323–1.918	<0.001	22.826 ± 1.762	1 *vs.* 2: <0.001;2 *vs.* 3: 0.064;1 *vs.* 3: <0.001
Group 2: SB							7.261 ± 0.530	
Group 3: Chemo							10.643 ± 1.556	
Body mass index								
Group 1: <18.5	0.662	0.508– 0.863	0.002	0.772	0.599–0.994	0.044	10.302 ± 1.863	1 *vs.* 2: 0.015;2 *vs.* 3: 0.079;1 *vs.* 3: 0.003
Group 2: 18.5–23.9							15.940 ± 1.441	
Group 3: >24							21.493 ± 2.687	
CA 19-9								
Group 1: <37	1.498	1.211–1.852	<0.001	1.325	1.064–1.651	0.012	25.287 ± 3.294	1 *vs.* 2: 0.042;2 *vs.* 3: 0.072;1 *vs.* 3: <0.001
Group 2: 37–200							15.387 ± 1.570	
Group 3: >200							12.471 ± 1.142	
Gender								
Group 1:Male	0.907	0.653–1.260	0.562	-	-	-	-	-
Group 2:Female								
Age								
Group 1:<65	1.133	0.813–1.581	0.460	-	-	-	-	-
Group 2: ≥65								
Albumin								
Group 1: <35	0.676	0.452–1.010	0.056	-	-	-	-	-
Group 2: ≥35								
Total bilirubin								
Group 1: <12	0.941	0.681–1.300	0.713	-	-	-	-	-
Group 2: ≥12								
Alanine Transaminase								
Group 1: <80	1.063	0.728–1.553	0.753	-	-	-	-	-
Group 2:≥80								
Carcinoembryonic antigen								
Group 1: <5	0.915	0.645–1.229	0.620	-	-	-	-	-
Group 2: ≥5								
CA 125								
Group 1: <35	1.365	0.998–1.886	0.059	-	-	-	-	-
Group 2: ≥35								
CA 50								
Group 1: <24	0.384	0.999–1.002	0.384	-	-	-	-	-
Group 2: ≥24								

Abbreviation: CA= Carbohydrateantigen, CI= Confidence interval, HR=Hazard ratio, PVR=Portal vein resection, SB=surgical bypass.

**Figure 2:**
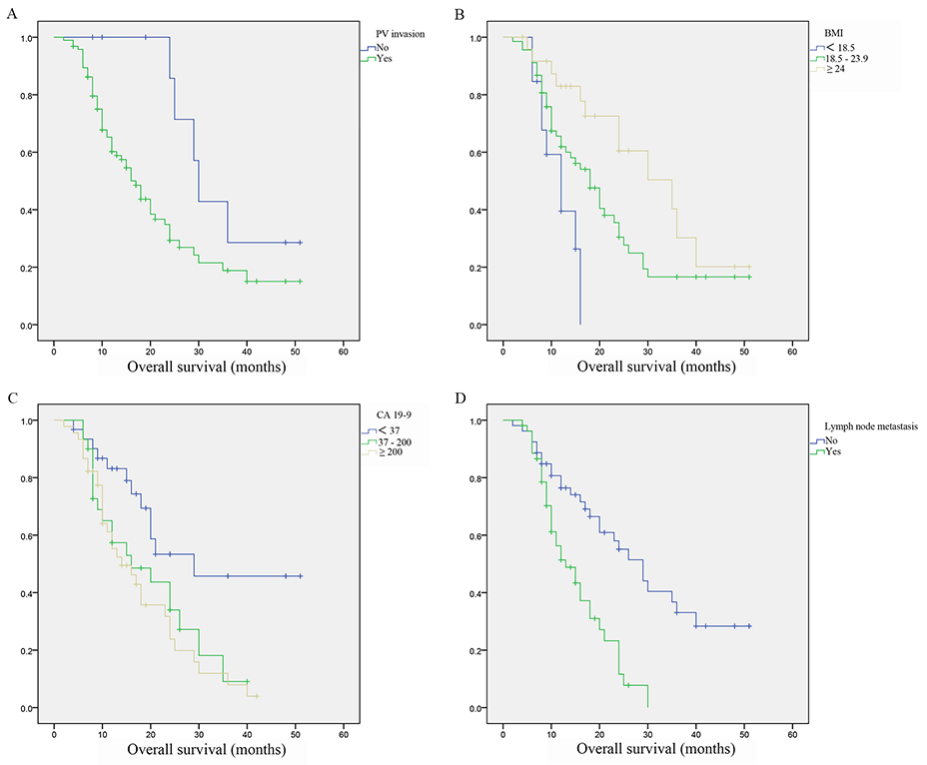
Survivals difference in pancreatic ductal adenocarcinoma patients with portal vein resection **(A)** Survivals difference in patients with or without portal vein invasion. **(B)** Survivals difference in patients with different body mass index. **(C)** Survivals difference in patients with different carbohydrateantigen 19-9 levels. **(D)** Survivals difference in patients with or without lymph node metastasis.

After performing the survival analysis among all PDAC patients, we then conducted univariate and multivariate regression analysis in patients with PVR in order to find out prognostic factors related with OS in patients with radical therapy. We found pathological PV involvement (HR =3.038, 95% CI 1.161 to 7.948, *P*=0.024), BMI (HR=0.582, 95% CI 0.374 to 0.904, *P*=0.016), CA 19-9 (HR=1.686, 95% CI 1.099 to 2.586, *P*=0.017) and lymph node metastasis (HR=2.541, 95% CI 1.455 to 4.438, *P*=0.001) were independent prognostic factors which significantly affected OS. Survival analysis were then performed with these risk factors and found patients with pathological PV involvement, worse BMI, higher CA 19-9 levels and lymph node metastasis achieved significantly worse survivals (Figure 2, Table 3).

**Table 3:** Multivariate analysis of risk factors related with overall survivals in patients with PVR

Risk factors	Multivariate analysis			Overall survival	
	HR	95% CI	P value	Mean ± SD, months	P value
Pathological PV invasion					
Group 1: Yes	3.038	1.161 – 7.948	0.024	21.485 ± 1.874	0.037
Group 2: No				35.143 ± 4.028	
Body mass index					
Group 1: <18.5	0.582	0.374 – 0.904	0.016	11.594 ± 1.157	1 *vs.* 2: 0.043;2 *vs.* 3: 0.039;1 *vs.* 3: <0.001
Group 2: 18.5 – 23.9				21.481 ± 2.137	
Group 3: >24				30.481 ± 3.480	
CA 19-9					
Group 1: <37	1.686	1.099 – 2.586	0.017	32.353 ± 3.863	1 *vs.* 2: 0.028;2 *vs.* 3: 0.531;1 *vs.* 3: 0.002
Group 2: 37 – 200				19.619 ± 2.294	
Group 3: >200				17.747 ± 1.837	
Lymph node metastasis					
Group 1: Yes	2.541	1.455 – 4.438	0.001	15.117 ± 1.167	<0.001
Group 2: No				29.077 ± 2.639	

Abbreviation: CA= Carbohydrateantigen, CI= Confidence interval, HR=Hazard ratio, PV=Portal vein, PVR=Portal vein resection.

### Surgery and Surgical complications

Together 158 patients underwent surgical procedure, 106 patients underwent PD with synchronous PVR and another 52 underwent SB. Compared with patients in SB group, patients in PVR group suffered significantly longer operation time (478.14 mins *vs.* 213.50 mins, *P*<0.001), more blood loss (600 ml *vs.* 120 ml, *P*<0.001), and longer hospital stay (21.02 days *vs.* 12.37 days, *P*<0.001). In patients underwent pancreaticoduodenectomy (PD) combined with PVR, 4.7% (5/106) of patients were found R1 resection. Moreover, pathological evidence from 10 patients (9.4%) in PVR group were found without PV involvement while other patients were all with pathological PV involvement.

No operation related death occurred and no in-hospital death were observed. During the hospital stay, no serious complications which threaten life or needing secondary operation occurred. Patients in PVR group suffered significantly higher incidence of pancreatic fistula, pleural effusion (Table 4). The difference of other complications between patients in PVR group and SB group were similar which showed PD combined with PVR was safe. B-ultrasonic test and computed tomography (CT) was required at every reexamination within postoperative 3 month to determine the condition of the interposition graft. Within postoperative 3 months, no graft infection and thrombosis was reported in patients in PVR group. Only 2 patients were observed postoperative bleeding during the hospital-stay.

**Table 4:** Postoperative complications in patients with portal vein resection or surgical bypass

Postoperative complications	PVR Group (n=106)	SB Group (n=52)	P value
Pancreatic fistula (A/B/C), n (%)	32 (30.2%) / 16 (15.1%) / 0 (0.0%)	1 (1.9%) / 0 (0.0%) / 0 (0.0%)	<0.001
Delayed gastric empting, n (%)	6 (5.7%)	0 (0.0%)	0.179
Postoperative bleeding, n (%)	2 (1.9%)	0 (0.0%)	>0.999
Wound infection, n (%)	8 (7.5%)	2 (3.8%)	0.499
Pleural effusion, n (%)	17 (16.0%)	2 (3.8%)	0.035
Abdominal infection, n (%)	2 (1.9%)	0 (0.0%)	>0.999

Abbreviation: PVR=Portal vein resection, SB=surgical bypass.

### Literature review

Together 13 studies from 8 countries satisfied our include criteria [11, 15, 17, 18, 20, 21, 25–31]. In these 13 studies, study sample varied from 78 patients to 1070 patients. We found there were 2 studies [25, 30] only enrolled stage II PDAC patients, however, enrolled patients in other studies varied from stage I to IV. Sample size of patients with PVR in 5 studies [11, 15, 16, 28, 30] was more than 100, while other studies were with a small sample size. Together 6 studies [11, 18, 21, 25–27] used both self-anastomosis (end to end anastomosis, suture, patch, autograft) and prosthesis (allograft), and other 6 studies [15, 17, 28–31] used self-anastomosis (Table 5).

**Table 5:** A systematic review of published studies

Study	Year	Country	PVR	Patients, n	Anastomosis	Tumor stage: 0/I/II/III/IV	Survival	Risk factors related with OS
Bachellier *et al.*	2001	France	Yes	21	S, P	0/0/5/4/12	1-year survival: 53.6%; 2-year survival:21.5%	N/A
			No	66	N/A	1/7/12/30/16	1-year survival: 51.1%; 2-year survival: 24.3%	
Chakravarty *et al.*	2010	China	Yes	12	S, P	0/0/12/0/0	1-year survival: 50.0%; 3-year survival:16.7%	Bilirubin, tumor differentiation, and adjuvant chemotherapy
			No	75	N/A	0/0/75/0/0	1-year survival: 44.4%; 3-year survival:12.2%	
Cheung *et al.*	2004	China	Yes	32	S, P	0/5/26/1/0	1-year survival: 70.6%; 3-year survival: 22.2%	Disease stage
			No	46	N/A	0/6/39/1/0	1-year survival: 71.1%; 3-year survival: 13.5%	
Fils *et al.*	2016	Slovenia	Yes	22	S, P	T_3_:22	Median OS: 16.2 mons	N/A
			No	111	N/A	T_1_:10, T_2_:26, T_3_:75	Median OS: 15.1 mons	
Gong *et al.*	2013	China	Yes	119	S, P	0/4/78/35/2	1-year survival: 30.0%; 3-year survival: 8.1%	The degree of tumor differentiation and the occurrence of complications after surgery
			No	447	N/A	0/36/84/3/0	1-year survival: 55.1%; 3-year survival: 21.2%	
Hartel *et al.*	2002	Germany	Yes	68	S, P	0/0/6/6/56	5-year survival: 23%	N/A
			No	203	N/A	0/27/50/124/2	5-year survival: 24%	
Hwang *et al.*	2015	Korea	Yes	147	S	T_1_:4, T_2_:9, T_3_:363, T_4_: 20	Median OS: 17.2 mons	The extent of venous involvement, LNM, and adjuvant chemotherapy
			No	396	N/A	T_1_:2, T_2_:3, T_3_:136, T_4_: 6	Median OS: 21.0 mons	
Mierke *et al.*	2016	Germany	Yes	113	N/A	T_1_+T_2_:4, T_3_+T_4_:109	Median DFS: P^+^I^+^, 7.4 mons; P^+^I^-^, 10.9 mons	True PV/SMV invasion
			No	66	N/A	T_3_+T_4_:66	Median DFS: 11.6	
Murakami *et al.*	2013	Japan	Yes	61	S	T_1_+T_2_:1, T_3_:61	Median OS: 14.7 mons	Adjuvant chemotherapy
			No	64	N/A	T_1_+T_2_:7, T_3_:57	Median OS: 26.7 mons	
Murakami *et al.*	2015	Japan	Yes	435	S	T_1_+T_2_:13, T_3_+T_4_:422	Median OS: 18.5 mons	Preoperative resectability status, CA19-9, blood transfusion, postoperative complications, LNM, and tumor stage
			No	502	N/A	T_1_+T_2_:69, T_3_+T_4_:433	Median OS: 25.8 mons	
Ouaissi *et al.*	2010	Belgium	Yes	59	S	0/20/58/0/4	Median OS: 17.5 mons	CA19-9, combined venous resection, and LNM
			No	82	N/A	0/10/37/11/1	Median OS: 18.7 mons	
Ravikumar *et al.*	2014	England	Yes	230	S	0/0/230/0/0	Median OS: 18.2 mons	N/A
			No	840	N/A	0/0/840/0/0	Median OS: 18.0 mons	
Shimada *et al.*	2006	Japan	Yes	86	S	0/0/47/0/39	Median OS: 14 mons	CA19-9, tumor size, serosal invasion, duodenal invasion, PV invasion, extra-pancreatic nerve plexus invasion, LNM, PVR, cancer infiltration at surgical margins, and intraoperative radiation therapy
			No	53	N/A	0/2/46/0/5	Median OS: 35 mons	

Abbreviation: CA=carbohydrate antigen; DFS=disease free survival; LNM= lymph node metastasis; N/A=not available; OS=overall survival; P^+^I^+^= PV resection and with PV infiltration; P^+^I^-^= PV resection and without PV infiltration; PVR=portal vein resection; P=prosthesis (allograft); S=self-anastomosis (end to end anastomosis, suture, patch, autograft); SMV=superior mesenteric vein.

In survival analysis, we found 7 studies [11, 15–17, 28, 29, 31] showed patients with PVR suffered from a worse OS than patients without PVR. However, rest 6 studies found the survival was similar between patients with or without PVR [18, 21, 25–27, 30]. A recently published meta-analysis [14] also showed one-, 3- and 5-year survival were worse in the PV- superior mesenteric vein (SMV) resection group. However, median overall survival was similar between 2 groups (14.3 months *vs.* 19.5 months, *P* = 0.063).

## DISCUSSION

For PDAC patients, pancreatic surgery provides the only chance for cure. However, only 20% of PDAC patients are eligible for surgery [32]. PV involvement used to be the contradictions for pancreatic surgery. At present, patients with PV involvement already have got the chance to be cured since the great improvement of the techniques and perioperative management. Currently, almost all studies are focusing on the benefits of PVR and few studies focused on how to choose the appropriate treatment for patients with PV involvement. At present, studies enrolled patients in all tumor stage, and pay little attention on patients with PV involvement. Those studies found out that PD combined with PVR only provided more chance for resection but did little to OS. Thus, we conducted the present study and patients we enrolled were all with PV involvement to solve such controversial issues. In the present study, we found out that patients received PD combined with PVR achieved a better OS compared with patients with SB and chemotherapy. PD combined with PVR is a safe and feasible therapy for patients with PV involvement.

Lots of studies [11, 28, 29] and newly published meta-analysis [14] claimed that patients with PVR had significantly worse survival. However, we found patients with PVR achieved the longest OS (mean survival: PVR, 22.83 months; SB, 7.26 months; Chemo, 10.64 months). This difference may due largely to the characeristics of enrolled populations. We only enrolled PDAC patients with PV involvement in this study in order to find out an appropriate therapy for them. In Shimada *et al.*'s study [31], patients underwent PVR had significantly worse OS than patients without (median survival: 14 months *vs.* 35 months). In their PVR group, 45.3% (39/86) of patients were with extra-pancreatic metastasis (tumor stage IV), however, there were only 9.4% of patients in non-PVR group were tumor stage IV. Based on such incomparable baseline characteristics, it is no wonder that patients in PVR group suffered significantly worse OS. In Ravikumar *et al.*'s study [30], they only enrolled stage II PDAC patients and found median survival was 18 months for patients with PD, 18.2 months for patients with PD combined with PVR, and 8 months for patients with SB. Compared with our findings (mean survival: PVR, 22.83 months; SB, 7.26 months; Chemo, 10.64 months), the result was similar. According to TNM classification stage, PV involvement and peri-pancreatic involvement were both classified as stage II. In patients with peri-pancreatic involvement PVR in no more needed, thus, they could set a group which only needed PD. However, in patients with PV involvement, PVR was the only choice for radical resection. That is why we only have 3 groups (PVR group, SB group and chemotherapy group) in our study.

In this study, we used artificial blood vessels (ePTFE vascular grafts) for reconstruction. In our literature review, there were 6 studies [11, 18, 21, 25–27] used both self-anastomosis and prosthesis (allograft), and other 6 studies [15, 17, 28–31] used self-anastomosis. Usually, the choice of reconstruction method should satisfied following criteria: invaded blood vessels are less than 1/3 of the circumference and with less severe involvement, suture and patch is used; invaded blood vessels are greater than 1/3 of the circumference and less than 5 cm, venous end-to-end anastomosis was used; and invaded blood vessels are longer than 5 cm, artificial blood vessels are commonly used. However, the severity of the invaded PV are based on intraoperative judgments of the operators. In this study, artificial blood vessels were used in order to achieve a radical resection and a negative vessel margin. Likely, ePTFE grafts has been used to secure tumor-free margins for patients in whom tumor-free margins cannot be obtained in liver surgery [33]. Unlike Self-anastomosis (end to end anastomosis, suture, and patch) are more likely to raise high tension of the anastomosed PV and may develop portal hypertension. Using ePTFE would reduce this risk. Compliance, lack of thrombogenicity, and resistance to infections as well as the ability to heal, remodel, contract, and secrete normal blood vessel products are theoretical advantages of artificial blood vessels [23, 24]. However, use of an artificial vascular graft has the potential risk of infection and thrombosis. In the use of ePTFE graft in liver surgery, no graft infection were reported [34, 35]. Moreover, ePTFE grafts are considered to have strong resistance to infection compared with other artificial vascular grafts [36]. The potential risk of luminal thrombus of the use of the ePTFE graft in low-flow vessels might be high. Compared with autogenous vein, ePTFE graft showed very high patency rates. The resistance of graft collapse, also resulting in few cases of graft thrombosis development [37]. In our study, patients with PVR were all asked to take aspirin and have ultrasonography examinations. Up till now, no graft infection and thrombosis were reported. However, in our study, the comparison of the benefits between autograft and allograft were not available. If it is possible, we should perform the study with such comparison.

Multivariate analysis showed different therapy choice, BMI and CA 19-9 levels were independent risk factors which influence OS. Patients with PVR suffered significantly longest survivals (mean survival: 22.83 months) may due to the patients with PVR were with resectable primary tumors. In patients with unresectable tumors, surgery would be not appropriate because it would do damage to immune system which may help produce tumor metastasis [38]. However, survivals between patients with SB and patients with chemotherapy were similar (mean survival: SB, 7.26 months; Chemo, 10.64 months, *P*=0.064) showed that different therapy would do little to influence the survivals in these patients. Also, patients with BMI less than 18.5 suffered significantly worst survivals. Take a further look at baseline characteristics, BMI was similar among 3 groups while patients in chemotherapy group suffered a significantly lower albumin (ALB) levels. ALB reflects physical nutrition status in some extent [39]. The prognosis would not be good when patients suffered negative nitrogen balance. Patients with BMI less than 18.5 were classified as underfat and suffered worse nutrition status. Thus, it is no wonder that patients with BMI less than 18.5 achieved worse survivals. In our study, higher CA 19-9 levels were associated with worse survival. Likely, Murakami *et al.* [28] and Shimada *et al.* [31] found CA 19-9 levels were independent risk factor of survivals. In pancreatic cancer, CA 19-9 levels were strongly correlate with tumor burden which may contribute to prognosis [40]. Thus a higher level of CA 19-9 correlated with a worse survival. Furthermore, fellow survival analysis conducted in patients with PVR also revealed lower BMI and higher CA 19-9 levels were correlated with worse OS which convinced our previous results. Interestingly, the survival analysis conducted in patients with PVR found pathological PV involvement and lymph node metastasis were also prognostic factors. Patients enrolled in our study were all with PV involvement based on initial computed tomography (CT) or magnetic resonance imaging (MRI) evidence. However, the status of PV involvement still needs postoperative pathological evidence. Tumor cells invaded into vascular wall may have a higher risk of developing distal metastasis. Thus a survival difference could be detected between patients with or without pathological PV involvement. Furthermore, patients with lymph node metastasis were classified as tumor stage IIb while patients with simply PV involvement were classified as tumor stage IIa. It was no wonder that patients with a late tumor stage achieved a worse OS.

The biggest limitation in our study is the retrospective study design. We already tried our best to enlarge our sample size. And we are the only study to specifically investigate the appropriate therapy for PDAC patients with PV involvement. At present, the prospective study design referring this issue is not available. Secondly, we are lack of the comparison between patients with autograft and allograft. We will perform the comparison in our future study.

In conclusion, resectable PDAC patients with PV involvement should choose PVR as their first-line treatment.

## MATERIALS AND METHODS

### Ethics statement

This study was approved by the Institutional Review Board of Shanghai Medical College of Fudan University (Shanghai, China), and conducted in accordance with the Declaration of Helsinki and internationally accepted ethical guidelines. The use of human tissue samples and clinical data was approved by the Clinical Research Ethics Committee of Huashan Hospital affiliated to Fudan University. All donors provided written informed consent to donate their samples. All methods were taken in accordance with the approved guidelines of Shanghai Medical College of Fudan University.

### Data availability

All data generated or analyzed during this study are included in this published article.

### Patients

From 2010 to 2015, there were 1382 consecutive PDCA patients admitted to our hospital. To be included in our study, patients had to be (a) aged 18-80 years; (b) without other organ metastasis; (c) clinical diagnosed as having PV involvement; (d) Eastern Cooperative Oncology Group score 0-2 and (e) definitively diagnosed with PDCA based on pathologic evidence. Patients were excluded from the study if they (a) had a history of therapy for PDAC; (b) had arterial involvement; (c) had other malignant tumors or extra-pancreatic metastasis.

Within one week before pancreatic surgery, all patients underwent a baseline assessment of serum leukocyte count, serum levels of alanine aminotransferase (ALT), total bilirubin (TBil), ALB; serum levels of carcinoembryonic antigen (CEA), CA125, CA 19-9 and CA 50. All patients were examined by contrast-enhanced CT or MRI, CT angiogram and positron emission tomography-computed tomography (PET-CT) scanning.

### Diagnosis of portal vein involvement

Based on preoperative imaging such as CT or MRI, we determined a high suspicion of PV involvement according to Nakao *et al.* [41]. Moreover, the possibility of PV involvement was then based on the surgeon's intraoperative visual judgment. Of course, the final evidence of PV involvement were clarified by postoperative pathological diagnosis.

### Therapy choice

**Figure 3:**
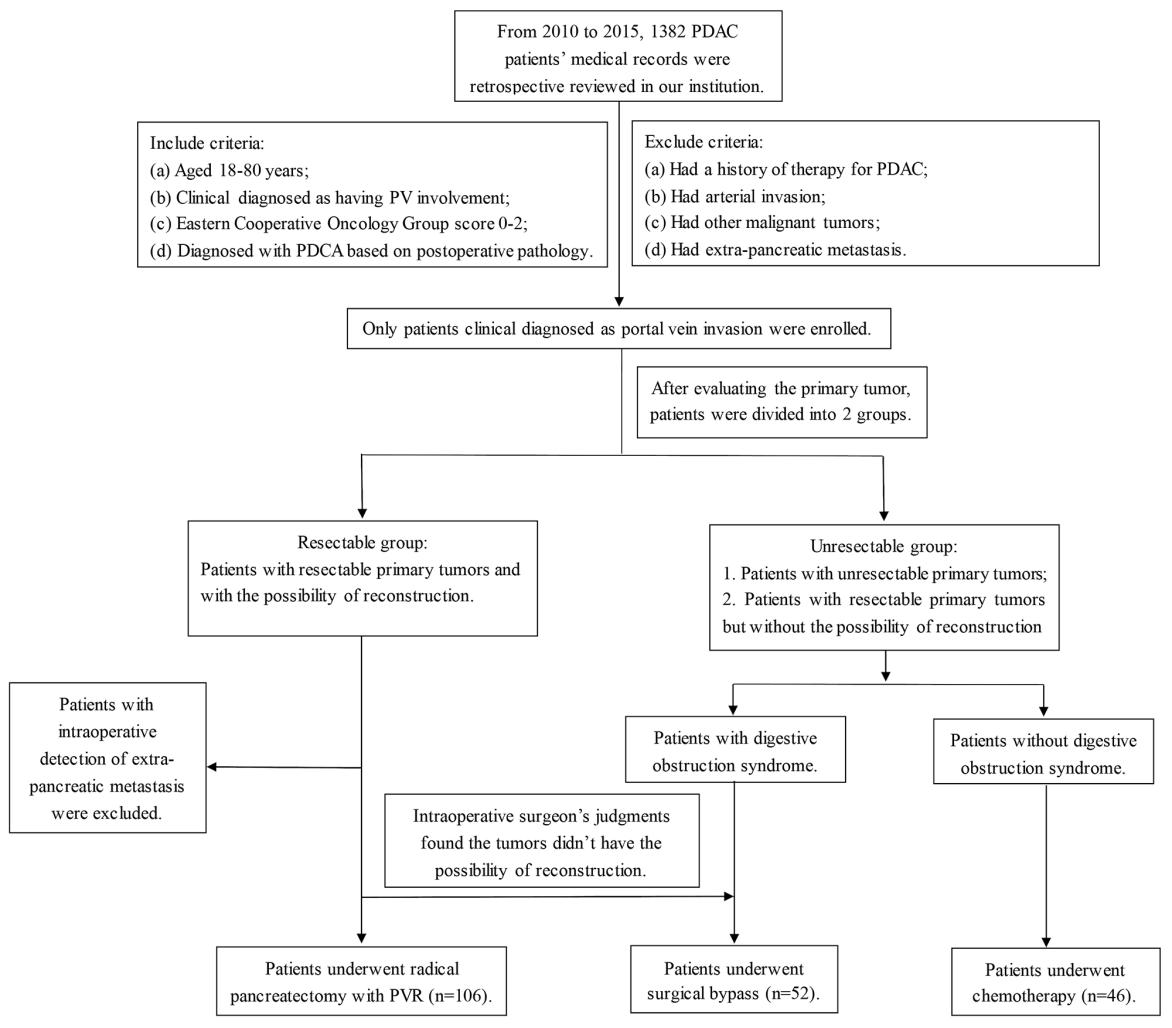
Selection flow *Notes:* From 2010 to 2015, 1382 potential eligible PDAC patients were enrolled in this study. Altogether 209 patients satisfied the inclusion and exclusion criteria (PVR group, n=111; SB group, n=50; chemo group, n=46). Three patients in PVR group were found liver metastasis during intraoperative exploration and were excluded. Tumors of two patients in PVR group were without the ability of reconstruction, thus undergoing SB procedure and were divided into SB group. Finally 204 eligible PDCA patients were enrolled (PVR group, n=106; SB group, n=52; chemo group, n=46).

We divided PDAC patients into resectable group or unresectable group according to initial examinations of pancreatic tumors by CT or MRI. Patients with resectable PDAC and had the possibility for reconstructions were divided into resectable group. In resectable group, all patients without operation contradictions were undergoing PD and PVR (PVR group). During the operation, we also used intraoperative ultrasound and surgeon's own visual judgments to evaluate the resectability of the primary tumor. Patients with any extra-pancreatic metastasis which were not detected by preoperative PET-CT scanning underwent SB procedure and were excluded from our study. Based on intraoperative surgeon's judgments, patients with PV involvement in resectable group but without possibility of reconstruction (multiple branch involvement) were divided into unresectable group and underwent SB procedure. Patients with unresectable tumors were treated with SB choice if patients were suffered from obstructive symptoms (SB group). For rest patients, we first used endoscopic ultrasound guided fine needle aspiration (FNA) biopsy for the confirm of the diagnosis of PDAC, followed by chemotherapy (chemotherapy group) (Flow was shown as Figure 3).

### Surgical procedures

For patients with resectable tumors, PD with PVR was performed. PD was performed a classic Whipple procedures. The Whipple's procedure commonly performed was PD with end-to-side pancreaticojejunostomy, end-to-side hepaticojejunostomy, and anterior-colic gastrojejunostomy. Two drains were placed at the end of the procedure in the foramen of Winslow and along with upper and below edge of the pancreaticojejunostomy. PVR were carried out en-bloc as primary closure of the vein, and reconstructed with ePTFE vascular grafts (Bard Peipheral Vascular, Inc).

**Figure 4:**
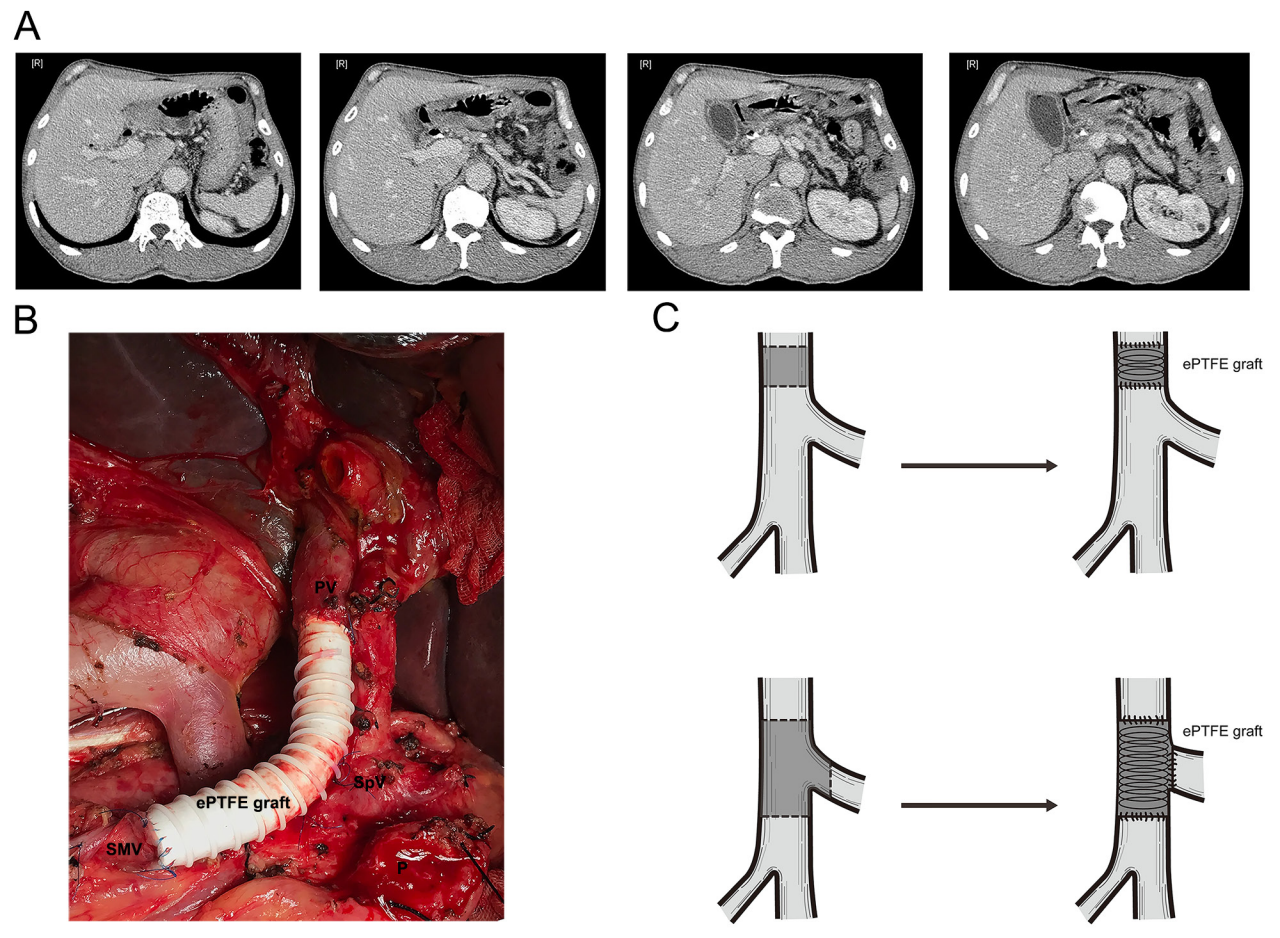
Schema of surgical procedures **(A)** CT scan of PDAC patients with PV involvement. **(B)** Surgical procedure of PD combined with PVR. **(C)** Sketch map of PD combined with PVR. *Abbreviation*: CT= Computer tomography, PD= pancreaticoduodenectomy, PDAC= pancreatic ductal adenocarcinoma, PV= Portal vein, PVR=Portal vein resection.

For patients with unresectable tumors, SB (hepaticojejunostomy with or without gastrojejunostomy) was performed. Intraoperative FNA was also conducted to provide the pathological evidence of the diagnosis of PDAC (Schema of surgical procedures were shown as Figure 4).

### Chemotherapy

In this study, the chemotherapy used in chemo group or used as the adjuvant therapy after surgery (both PD and SB) was intra-arterial infusion chemotherapy (IRIC) [42]. We placed 5-Fr Rosch hepatic catheters and used Seldinger′s technique via the femoral artery. We reconfirmed the position by digital subtraction angiography (DSA) with the tip into the hepatic artery or the superior mesenteric artery (SMA). The mixture of 5-fluorouracil (600 mg/m^2^), cisplatin/oxaliplatin (30 mg/m^2^) and gemcitabine (1000 mg/m^2^) was then injected into SMA. Any patients received chemotherapy were given one cycle every 4 weeks for 6 cycles.

### Postoperative management

Patients were required to reexamine every 4 weeks. The same serum tests as baseline were tested. An increase of serum CA 19-9 levels with the abnormal mass of the residue pancreas discovered by CT or MRI was defined as recurrence. In PVR and SB group, patients were given IRIC one cycle every 4 weeks for 6 cycles.

For patients underwent PD with PVR, aspirin 50-100 mg per day were given. And B-ultrasonic test was required at every reexamination to determine the condition of the interposition graft.

### Outcomes

At baseline, body mass index (BMI), leucocytes, ALB, TBil, ALT, CEA, CA 125, CA 19-9, CA 50 were compared among 3 groups. Tumor diameters, operation time, blood loss were recorded intra-operatively and compared between 2 surgical groups. Primary outcomes were OS, and secondary outcomes were surgical complications.

Pancreatic fistula (PF), delayed gastric emptying (DGE) and intra-abdominal hemorrhage were defined according to the International Study Group of Pancreatic Surgery [43, 44]. Intra-abdominal infection was defined according to the Centers for Disease Control and Prevention [45]. Mortality was defined as any death that occurred in the 30 days after surgery or during the hospital stay.

### Statistical analysis

The soft SPSS 21.0 (IBM, Chicago, USA) was used for statistical analysis, and P < 0.05 was defined as the threshold of statistical significance. Normally distributed data was expressed as mean ± standard deviation (SD), while asymmetrically distributed data was expressed as median (range). Differences in outcomes among PVR group, SB group and chemo group were assessed for significance using ANOVA tests. Kaplan–Meier method was used to calculate survival curves. Multivariate survival analysis was performed by Cox proportional hazards model.

### Literature review

We also conducted a literature review, systematically reviewed Pubmed, web of science, and Cochrane Library using following key words: “*pancreatic cancer*” OR “*pancreatic ductal adenocarcinoma*”; “*portal vein*”; “*resection*” OR “*surgery*” to evaluate the benefit of radical pancreatectomy with PVR. Studies enrolled in this review should (a) have at least 2 arms (PVR *vs.* not-PVR); (b) carefully described the tumor stage of enrolled patients; (c) reported survival data.

## Abbreviations

ALB: albumin
ALT: alanine aminotransferase
BMI: body mass index
CA: carbohydrateantigen
CEA: carcinoembryonic antigen
CI: confidence interval
CT: computed tomography
FNA: fine needle aspiration
HR: hazard ratio
IRIC: intra-arterial infusion chemotherapy
MRI: magnetic resonance imaging
PD: pancreaticoduodenectomy
PDAC: pancreatic ductal adenocarcinoma
PET-CT: positron emission tomography-computed tomography
PV: portal vein
PVR: portal vein resection
SB: surgical bypass
TBil: total bilirubin
